# Recent Progress in Flexible and Wearable All Organic Photoplethysmography Sensors for SpO_2_ Monitoring

**DOI:** 10.1002/advs.202302752

**Published:** 2023-09-23

**Authors:** Jostin Vinroy Dcosta, Daniel Ochoa, Sébastien Sanaur

**Affiliations:** ^1^ Mines Saint‐Étienne Centre Microélectronique de Provence Department of Flexible Electronics 880, Avenue de Mimet Gardanne 13541 France

**Keywords:** flexible organic devices, organic biosensors, organic light emitting diodes, organic photodetectors, SpO_2_ sensors, photoplethysmography, pulse oximeter

## Abstract

Flexible and wearable biosensors are the next‐generation healthcare devices that can efficiently monitor human health conditions in day‐to‐day life. Moreover, the rapid growth and technological advancements in wearable optoelectronics have promoted the development of flexible organic photoplethysmography (PPG) biosensor systems that can be implanted directly onto the human body without any additional interface for efficient bio‐signal monitoring. As an example, the pulse oximeter utilizes PPG signals to monitor the oxygen saturation (SpO_2_) in the blood volume using two distinct wavelengths with organic light emitting diode (OLED) as light source and an organic photodiode (OPD) as light sensor. Utilizing the flexible and soft properties of organic semiconductors, pulse oximeter can be both flexible and conformal when fabricated on thin polymeric substrates. It can also provide highly efficient human‐machine interface systems that can allow for long‐time biological integration and flawless measurement of signal data. In this work, a clear and systematic overview of the latest progress and updates in flexible and wearable all‐organic pulse oximetry sensors for SpO_2_ monitoring, including design and geometry, processing techniques and materials, encapsulation and various factors affecting the device performance, and limitations are provided. Finally, some of the research challenges and future opportunities in the field are mentioned.

## Graphical abstract

The need for continuous, non‐invasive and real‐time monitoring of cardiovascular vital signs is of primary importance. This requires human‐friendly wearable sensor systems that can be easily and conformably integrated onto the human body. Organic electronic devices may fulfill all those properties. Here, the recent biosensing progress in flexible and wearable all‐organic SpO_2_ monitoring devices is reviewed.

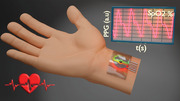

## 1 Introduction

### 1.1 Pulse Oximeter as Flexible and Wearable Organic PPG Sensor

With the increase in cardiovascular‐related mortality rate every year,^[^ ^1^ ^]^ the need for continuous, non‐invasive and real‐time monitoring of cardiovascular vital signs such as heart rate (HR), blood pressure (BP), SpO_2_, and blood glucose has become significant as most of the cardiovascular‐related issues can be prevented if diagnosed at an early stage.^[^ ^2^, ^3^, ^4^ ^]^ Moreover, the continuous and real‐time monitoring of these vital signs requires efficient and human‐friendly wearable sensor systems that can be easily and conformably integrated with the human body without interfering with daily activities. Several sensor mechanisms exist that can efficiently monitor various cardiovascular vital signs from different body‐sensing locations. They include bioelectric, mechano‐electric, electrochemical, optoelectronic, and ultrasonic sensors.^[^ ^5^ ^]^ For example, heart rate can be obtained by optoelectric, mechano‐electric, or ultrasonic type sensors utilizing the pulsatile signal waveforms obtained from ECG,^[^ ^6^ ^]^ photoplethysmography (PPG),^[^ ^7^ ^]^ seismocardiography^[^ ^8^ ^]^ ballistography^[^ ^9^ ^]^ and ultrasonography.^[^ ^10^ ^]^ SpO_2_ can be obtained by monitoring the PPG signals from the subcutaneous arteries using optoelectronic sensors.^[^ ^11^ ^]^ Blood glucose can be monitored by an electrochemical sensing mechanism via saliva, sweat, tears, and interstitial fluids.^[^ ^12^, ^13^ ^]^

Among the various biosensors for cardiovascular monitoring, optoelectronic biosensors take advantage of the straightforward changes in the optical properties of the different biometric elements, due to light absorption, reflection, transmission, interference, emission, or fluorescence patterns. The sensors usually consist of photodetectors that quantify the optical changes and convert them into their corresponding electrical signals. These electrical signals are then used to evaluate vital signs such as heart rate, respiration rate, and SpO_2_.^[^ ^14^, ^15^ ^]^ A classic example of optoelectronic biosensor is a pulse oximeter that takes advantage of variable light absorption characteristics of the oxygenated (HbO_2_) and de‐oxygenated (Hb) hemoglobin in blood volume and does a ratiometric optical measurement at two different wavelengths to evaluate the percentage oxygen saturation.^[^ ^16^ ^]^ (the working mechanism of the pulse oximeter is explained in Section 3 of the review).

Pulse oximeter sensor monitors the oxygen saturation in an individual, as it is an important vital sign that gives information on the adequacy of the respiratory function. The normal SpO_2_ in a healthy individual should be at least 95%.^[^ ^17^ ^]^ The conventional oxygen saturation test is performed invasively by drawing blood from an artery. It is called an arterial blood gas test (ABG) and is usually termed as SaO_2_.^[^ ^18^, ^19^ ^]^ Unlike this invasive ABG technique, the non‐invasive oxygen saturation measurement uses the pulse oximetry technique and is termed as peripheral oxygen saturation or SpO_2_.^[^ ^20^, ^21^ ^]^ Usually, the calibration of the SpO_2_ sensor is done by comparing the oxygen saturation values with the values obtained from SaO_2_ signals from healthy individuals. An accuracy of 2%, evaluated by the standard deviation of the difference between SaO_2_ and SpO_2_ is accepted globally.^[^ ^22^ ^]^

Pulse oximeter sensors also play a significant role in detecting early stages of tissue deterioration,^[^ ^23^ ^]^ blood circulation disorders,^[^ ^24^ ^]^ and lung issues.^[^ ^25^ ^]^ One of the common applications of pulse oximeter is monitoring patients who are at risk of hypoxia or low blood oxygen level conditions.^[^ ^26^, ^27^ ^]^ Hence, the sensor can be usually found in operating rooms, intensive care units, and neonatal caring units where continuous monitoring of oxygen saturation is essential. Another important application of pulse oximeter sensor is in monitoring SpO_2_ levels in patients who are placed under artificial ventilation with supplemental oxygenation.^[^ ^28^, ^29^, ^30^, ^31^ ^]^ The sensor allows to better regulate and monitor oxygen concentration, thereby avoiding over‐oxygenation.

With the disparate mechanical properties of human body such as curvilinear, dynamic deformation, and softness, achieving body conformal electronics using commercially available conventional planar and rigid components and interfaces is challenging.^[^ ^32^ ^]^ Commercial pulse oximeter sensors are usually clipped on a fingertip and consist of a rigid in‐organic semiconductor material such as silicon as an active sensing element and commercially available LEDs as light source. Moreover, the wearable and body conformal applications of silicon‐based electronics are limited due to their bulkiness, rigidity, and large area scaling cost.^[^ ^33^ ^]^ In addition to this, the continuous deformation of bodily skin due to even minor movements can hinder the accurate measurement of data. For example, movements in the body joints can cause skin deformation up to 60%^[^ ^34^ ^]^ and hence can result in a decrease of the SNR during pulse oximetry measurements. For this reason, commercially available wearables such as smart watches,^[^ ^35^, ^36^ ^]^ and wrist bands^[^ ^37^, ^38^ ^]^ for SpO_2_ monitoring with miniaturized inorganic components have been already developed for better sensor functionality and are on the cusp in the electronic consumer market. However, these wearables are still rigid, always located in the same place on the body, still lack local point conformability, and hence limit the accuracy of data when interfaced with a flexible skin. Hence, there is a need for a better alternative that is flexible and conformal for the better accuracy and data readability of sensors when integrated with deformable skin. On the other hand, advancements in material science and technology have enabled the development of soft, flexible, and stretchable sensors with miniaturized active components on flexible substrates that are no longer constrained to the planar geometries and have the potential to transmit accurate data when directly interfaced with flexible skin for smart bio‐signal monitoring.^[^ ^39^, ^40^ ^]^ Moreover, using organic semiconductor components for LEDs and PDs in pulse oximetry are more promising for providing accurate measurements in body conformal electronics than using conventional inorganic material‐based components in area‐comparable pulse oximeters^[^ ^41^, ^42^ ^]^ Thus parallel efforts are been made by researchers to fabricate flexible and conformal biosensors with organic semiconductors as active elements that are intrinsically soft and flexible by nature.^[^ ^43^, ^44^, ^45^ ^]^ Moreover, organic semiconductors with low‐cost solution processing abilities,^[^ ^46^, ^47^ ^]^ bio‐compatibility^[^ ^48^ ^]^ and light weightiness^[^ ^49^ ^]^ can be directly coated onto ultra‐thin,^[^ ^50^ ^]^ flexible^[^ ^51^, ^52^ ^]^ and conformal polymeric substrates.^[^ ^53^ ^]^ They are seamlessly integrated with large areas of curvy body surfaces and at the same time provide better local skin conformability, without having the constraint of miniaturization, unlike inorganic components. Hence with reduced complexity in processing, versatile chemistry^[^ ^54^ ^]^ and compatibility with arbitrary substrates, organic electronic devices have the possibility to replace conventional silicon in conformal biosensors applications.

Despite the advantages of pulse oximetry in biosensing, there are also challenges such as sensor location, temperature effect, motion artifact, pressure effect, optical loss due to refractive index mismatch between substrate and skin, low blood oxygen levels, skin pigmentation and presence of dyshemoglobinemias.^[^ ^55^, ^56^ ^]^ In addition to this, the organic layers in the OLEDs and OPDs tend to degrade with time.^[^ ^57^, ^58^ ^]^ Therefore, suitable encapsulation techniques for the better lifetime of the sensor are also a challenge. Further, at present pulse oximeters use inorganic material‐based analog frond end (AFE) rigid components including transistors and amplifiers to read the PPG signals. This limits the form factor minimization of the organic SpO2 sensor. Hence, to address these issues there is a need to develop novel pulse oximetry biosensing systems, with high efficiency, better output readability, and favorable form factors. Innovations in design strategies, stable and efficient optoelectronic materials, processing techniques for OLED and OPD, integration with system software and hardware are the bottleneck requirements to achieve highly efficient flexible, and all organic optoelectronic biosensing systems.

### 1.2 Techno‐Economic Analysis

The increase in chronic medical ailments across the globe along with rising geriatric population are the major key factors driving the growth of SpO_2_ sensor market. Cardiovascular diseases (CVDs) account for >31% of global death every year with >17 million fatalities and is expected to increase to 23.6 million by the year 2030.^[^ ^59^ ^]^ Despite the high mortality rate, ≈90% of CVDs can be prevented by early detection. According to a report from the World Health Organization (WHO) in 2022, chronic obstructive pulmonary disease (COPD) is the third leading cause of death worldwide and caused nearly 3.23 million deaths in 2019 and nearly 90% of the COPD deaths under 70 years of age occur in low and middle‐income countries.^[^ ^60^ ^]^ Alongside CVDs, the rising prevalence of the target disease such as asthma and related respiratory disorders,^[^ ^61^, ^62^ ^]^ hypertension,^[^ ^63^ ^]^ diabetes,^[^ ^64^ ^]^ ischemic disorders,^[^ ^65^, ^66^ ^]^ cardiac arrhythmia,^[^ ^67^ ^]^ hyperlipidemia,^[^ ^68^ ^]^ and sleep apnea^[^ ^69^, ^70^ ^]^ have further demanded the use of SpO_2_ sensor systems for continuous health monitoring. For example, pulse oximeter sensors have shown their utmost significance during the covid‐19 pandemic in monitoring the blood oxygen concentration in critically ill patients suffering from respiratory illness.^[^ ^71^, ^72^, ^73^ ^]^ According to the recent market report by IMARC group the global wearable market for SpO_2_ sensors has reached up to 2.3 billion US dollars for the year 2022 and is estimated to reach the market value of 3.5 billion by 2028, exhibiting a compound annual growth rate of 7%.^[^ ^74^ ^]^

At present, inorganic pulse oximeters (i.e., made from silicon‐based components) often require specific sensor locations on the human body for accurate measurements. Currently, the fingertip is the most typical location used for inorganic pulse oximeters available in the market.^[^ ^75^ ^]^ However, the location and bulkiness of the sensors and readout circuit for signal processing pose challenges in precise readings from individuals with reduced peripheral circulation, leading to erroneous results.^[^ ^76^ ^]^ Additionally, in certain patient populations such as newborns or individuals with short fingers, the size of the fingertip may not allow for proper sensor alignment leading to poor contact and inaccurate SpO_2_ readings.^[^ ^77^, ^78^ ^]^ Moreover, during physical activities that require frequent hand movements, continuous health monitoring may not be desirable as the sensor's position on the fingertip may become unstable leading to motion artifacts and inconsistent readings.^[^ ^79^, ^80^ ^]^

However, although bulky inorganic material‐based pulse oximeter sensors currently dominate the SpO_2_ sensor market, the requirement of flexible and wearable user‐friendly sensors in continuous health monitoring has demanded the growth of organic material‐based pulse oximeter sensor systems that can be seamlessly integrated onto the human body without restriction in the sensor location with new ways of health diagnostic and bioanalysis. Alongside, due to the inherent property of organic materials in OLEDs and OPDs, organic pulse oximeters consume relatively less power resulting in reduced energy requirements and extended battery life.^[^ ^81^ ^]^ Additionally, wearable organic pulse oximeters have the ability to incorporate energy harvesting technologies such as solar cells and can even eliminate the need for batteries thus offering sustainable and self‐powered operation.^[^ ^82^ ^]^ Furthermore, with increase in demand for the non‐invasive type of personal health care monitoring and growing health and fitness concerns among people together could accelerate the growth of organic pulse oximeter adoption.^[^ ^83^, ^84^ ^]^ Moreover, it is today recognized that their potential will completely transform and revolutionize patient monitoring as these sensors show improved accuracy since they can adapt to uneven skin surfaces and reduce motion aberrations.^[^ ^85^, ^86^, ^87^ ^]^ As consumers seek constant health monitoring, including SpO_2_ and heart beat count during diverse activities like exercise, work, sleep, and other daily activities, organic sensors provide a reliable and consumer‐friendly solution to these applications.

Despite organic pulse oximeter sensors are in the research stage, it is anticipated to expand and experience robust growth in the coming years. By combining the developments in organic electronics, material science, and wireless communication, serious realization has been made. Ongoing research and developmental efforts are focused to increase their durability, stability, and performance. Additionally, development of novel organic materials and fabrication techniques will likely lead to more reliable and accurate sensors. Moreover, the miniaturization of organic pulse oximeter sensors will enable integration on a variety of wearable electronic gadgets will allow to continuously and covertly check the oxygen saturation levels in a variety of environments. While organic pulse oximeter sensors offer numerous advantages, it is crucial to note that the technology is still in its early development stage and hence further research and refinement are required. Further, in order to ensure its reliability and safety for clinical use regulatory approvals, standardization, and validation studies are necessary. Incorporating smart features and systems such as easy data transfer, large memory, alarm systems, wireless communications via Bbluetooth, and artificial intelligence will further boost the market.^[^ ^88^, ^89^, ^90^ ^]^

## 2 Sensing Modes in Pulse‐Oximetry

**Figure 1:**
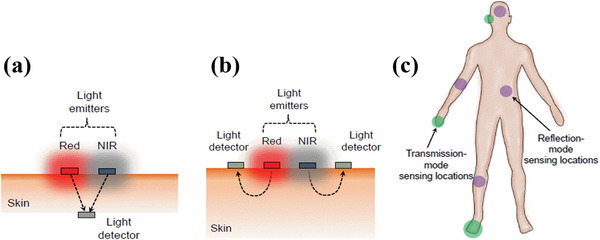
a) In the transmission mode oximetry, the photodetector is on the opposite side of red and NIR light emitters which are placed on the skin. b) In the reflection mode oximetry, the light detector is on the same side as red and NIR light emitters and all the components are placed on the skin. The arrows indicate the direction of light propagation through the skin reaching the photodetector. c) Body‐suitable sensing locations for oximetry. The green highlighted areas (fingers, earlobes, and feet) are suitable for operation in the transmission mode oximetry whereas sensing locations highlighted in purple (forehead, abdomen, fore arm, leg, etc.) are suitable for operation in the reflection mode oximetry. Reproduced with permission.^[^ ^94^ ^]^ Copyright 2018, National Academy of Sciences.

A pulse oximeter sensor can be operated either in the reflection mode or in the transmission mode. In the transmission mode, the light emitter is placed on one side of the sensing area and the light transmitted through the sensing area is detected by the detector placed on the opposite side, as shown in **Figure** **1**a. However, this type of operation restricts the sensing location as sensing can be performed only on the areas that can be trans‐illuminated, e.g., earlobes, fingers, and feet for neonates.^[^ ^19^, ^56^, ^91^ ^]^ On the other hand, reflection mode oximetry consists of a light emitter and detector both placed on the same side of the sensing area as shown in Figure 1b. In this sensing mode, the backscattered light from the area of interest is detected by the photodetector placed on the same side of the sensing area. This sensing mode is suitable for sensing locations such as forearm, leg, abdomen, forehead, etc., and is suitable at various body sensing locations.^[^ ^92^, ^93^ ^]^ The different sensing locations for pulse oximetry along with the sensing modes are shown in Figure 1c.

## 3 Operation Mechanism in Pulse Oximetry

**Figure 2:**
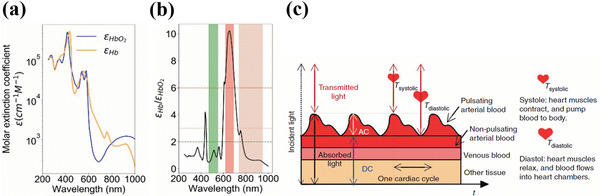
a) Molar extinction coefficients of Hb and HbO_2_. b) Ratio of molar extinction coefficients of Hb and HbO_2_. Reproduced with permission.^[^ ^94^ ^]^ Copyright 2018, National Academy of Sciences. c) Schematic illustrating the light transmission path through the pulsatile and non‐pulsatile arterial blood, venous blood, and tissues and generation of PPG signals consisting of DC and AC components over a few cardiac cycles during systolic and diastolic phases. Reproduced with permission.^[^ ^98^ ^]^ Copyright 2014, Springer Nature.

The molar absorptivity of Hb and HbO_2_ varies with the wavelength as shown in **Figure** **2**a. The two wavelengths for pulse oximetry measurements are chosen in such a way that there is sufficient contrast in the molar extinction coefficients of Hb and HbO_2_ at those wavelengths. Usually, a combination of red‐green or red‐NIR light wavelengths has adequate contrast.

The range of wavelength suitable for oximetry includes green (470–550 nm), red (620–690 nm), and NIR (740–950 nm).^[^ ^95^ ^]^ The corresponding ratio of absorption coefficients are$\left(\varepsilon_{Hb}/\varepsilon_{HbO_{2}}<2\right)$ for green, $\left(\varepsilon_{Hb}/\varepsilon_{HbO_{2}}>6\right)$ for red and ($\varepsilon_{Hb}/\varepsilon_{HbO_{2}}<3)$ for NIR, as shown in Figure 2b.^[^ ^94^ ^]^

In the pulse oximetry measurement setup, the incident light from the light source is attenuated by the pulsatile component of the arterial blood containing Hb and HbO_2_, non‐pulsatile arterial blood, venous blood, and other tissues as shown in Figure 2c. The PPG signals are generated because of light attenuation by the arterial blood during the systolic (heart contraction phase) and diastolic (heart relaxation phase) of the cardiac cycle. In the systolic cycle, the flow of blood into the arteries increases the arterial blood volume in the sensing location resulting in maximum light attenuation by Hb and HbO_2_. In the diastolic cycle, the attenuation minimum occurs due to the decrease in blood volume because of the reverse flow of blood into the heart. The light intensity reaching the PD after attenuation is amplified and converted into an electrical signal constituting the PPG. The generated PPG signal consists of rapidly changing alternating (AC) components due to the varying absorption of light wavelength by the pulsatile arterial blood and steady‐state direct (DC) component due to the absorption by non‐pulsatile components such as veins, capillaries, bones and also scattered light by tissues.^[^ ^96^, ^97^, ^98^, ^99^, ^100^ ^]^ The fundamental frequency of the AC component depends on the heart rate in an individual.

Usually, an OPD can sense a broad range of light spectrum and is combined with two OLEDs that act as sources of two different light wavelengths. With this configuration, the OPD itself cannot distinguish the two wavelengths if illuminated simultaneously. Hence, sequential sampling of the light at a given frequency is done during the measurement and the OPD is synched with the optoelectronic system software and hardware for good resolution of PPG signals. This way, the sequential sampling of the light intensity by the OPD after attenuation in the systolic and diastolic cycle gives information about the absorption characteristics of oxygenated and de‐oxygenated hemoglobin in the arterial blood. In the presence of the pulsatile signal, the SpO_2_ is the total concentration of HbO_2_ divided by the total sum of concentrations of Hb and HbO_2_ and can be analytically quantified according to the following,

(1) $$\begin{array}{c} SpO_{2}=\frac{C_{HbO_{2}}}{C_{HbO_{2}}+C_{Hb}}=\frac{\varepsilon_{\lambda_{1},Hb}-\varepsilon_{\lambda_{2},Hb}\;{R^{\prime }}_{os}}{\left(\varepsilon_{\lambda_{1},Hb}-\varepsilon_{\lambda_{1},\;HbO_{2}}\right)+\left(\varepsilon_{\lambda_{2,}HbO_{2}}-\varepsilon_{\lambda_{2},Hb}\right){R^{\prime }}_{os}} \end{array}$$

Where, $C_{HbO_{2}}$ and *C_Hb_* are the concentrations of HbO_2_ and Hb respectively. $\varepsilon_{\lambda_{1,}Hb}$ and $\varepsilon_{\lambda_{2},HbO_{2}}$ are the molar extinction coefficients of hemoglobin and de‐oxyhemoglobin at wavelengths λ_1_and λ_2_respectively.

(2) $$\begin{array}{c} R_{os}^{\prime }=\frac{R_{os}}{\frac{DPF_{\lambda_{1}}}{DPF_{\lambda_{2}}}},\;\mathrm{where}\;R_{os}=\frac{AC_{\lambda_{1}}/DC_{\lambda_{1}}}{AC_{\lambda_{2}}/DC_{\lambda_{2}}},\;\;\mathrm{is}\;\mathrm{the}\;\mathrm{modulation}\;\mathrm{ratio} \end{array}$$

DPF is the differential path length factor and the value depends on the optical path length of the reflected light and accounts for the multiple scattering of light in the tissue. $AC_{\lambda_{1}}/DC_{\lambda_{1}}$ and $AC_{\lambda_{2}}/DC_{\lambda_{2}}$ are the ratios of AC to DC signals received by the OPD at the incident wavelengths λ_1_ and λ_2_ respectively.

In the case where there is very low blood perfusion, the pulsatile signal from the arteries will be very weak, and hence a direct estimation of SpO_2_ either in the reflection or transmission mode oximetry cannot be performed. In that scenario the change in oxygen saturation $\left(\Delta \mathrm{Sp}O_{2}\right)$ can be evaluated by from Beer‐Lambert law by measuring the time‐varying light intensity attenuation (Δ*I*) as

(3) $$\Delta I_{\left(\lambda \right)}=I_{0_{\left(\lambda \right)}}\;e^{-\Delta \mu_{a_{\left(\lambda \right)}}.d.DPF_{\left(\lambda \right)}}$$

Where *I*_0_ is the incident light intensity, d is the OPD to OLED spacing distance and $\Delta \mu_{a_{\left(\lambda \right)}}$ is the change in light absorption during the measurement, and can be further expressed as the sum of absorption coefficients for HbO_2_ and Hb at a wavelength 𝜆 times the concentrations $\Delta C_{HbO_{2}}$ and $C_{Hb}$ as

(4) $$\Delta \mu_{a_{\left(\lambda \right)}}=\varepsilon_{HbO_{2_{\left(\lambda \right)}}}.\;\Delta C_{HbO_{2}}+\varepsilon_{Hb_{\left(\lambda \right)}}.\Delta C_{Hb}$$

Rewriting Equation (3):

(5) $$\varepsilon_{HbO_{2_{\left(\lambda \right)}}}.\Delta C_{HbO_{2}}+\varepsilon_{Hb_{\left(\lambda \right)}}.\Delta C_{Hb}=\frac{\ln\frac{I_{0_{\left(\lambda \right)}}}{\Delta I_{\left(\lambda \right)}}}{d.DPF_{\left(\lambda \right)}}$$

Equation (5) can be expressed as a system of two linear equations for two different wavelengths as

(6) $$\varepsilon_{HbO_{2_{\left(\lambda_{1}\right)}}}.\Delta C_{HbO_{2}}+\varepsilon_{Hb_{\left(\lambda_{1}\right)}}.\Delta C_{Hb}=\frac{\ln\frac{I_{0_{\left(\lambda_{1}\right)}}}{\Delta I_{\left(\lambda_{1}\right)}}}{d.DPF_{\left(\lambda_{1}\right)}}$$

(7) $$\varepsilon_{HbO_{2_{\left(\lambda_{2}\right)}}}.\Delta C_{HbO_{2}}+\varepsilon_{Hb_{\left(\lambda_{2}\right)}}.\;\Delta C_{Hb}=\frac{\ln\frac{I_{0_{\left(\lambda_{2}\right)}}}{\Delta I_{\left(\lambda_{2}\right)}}}{d.DPF_{\left(\lambda_{2}\right)}}$$

Equations (6) and Equation (7) can be rewritten to evaluate for the change in concentrations of Hb and HbO_2_ as

(8) $$\left[\begin{array}{cc} \varepsilon_{\left(\lambda_{1},HbO_{2}\right)} & \varepsilon_{\left(\lambda_{1},Hb\right)}\\ \varepsilon_{\left(\lambda_{2},HbO_{2}\right)} & \varepsilon_{\left(\lambda_{2}Hb\right)} \end{array}\right]\;\left[\begin{array}{c} \Delta C_{\left(HbO_{2}\right)}\\ \Delta C_{\left(Hb\right)} \end{array}\right]=\left[\begin{array}{c} \frac{\ln\frac{I_{0_{\left(\lambda_{1}\right)}}}{\Delta I_{\left(\lambda_{1}\right)}}}{d.DPF_{\left(\lambda_{1}\right)}}\\ \frac{\ln\frac{I_{0_{\left(\lambda_{2}\right)}}}{\Delta I_{\left(\lambda_{2}\right)}}}{d.DPF_{\left(\lambda_{2}\right)}} \end{array}\right]$$

The molar extinction coefficients^[^ ^101^, ^102^, ^103^ ^]^ and DPF^[^ ^104^, ^105^, ^106^, ^107^ ^]^ can be obtained from the literature. Solving Equation (8), $\Delta C_{HbO_{2}}$and Δ*C_Hb_* can then be evaluated.^[^ ^94^, ^98^ ^]^ Hence, by knowing the change in Hb and HbO_2_ concentrations during a transient measurement, Δ*SpO*_2_ can be determined from Equation (1).

Apart from SpO_2_, pulse oximetry PPG signals can also be used to monitor the heart rate (HR). With each heart beat oxygenated blood is pumped into the blood vessels, whereas deoxygenated blood returns back to the heart. This periodic flow is causing the subcutaneous arteries to expand and contract, and consequently modulate periodically the light absorptivity for Hb and HbO_2_. The HR refers to the number of heart beats per minute (bpm) and this can be estimated as

(9) $$HR=\frac{60}{T}\;\left(\mathrm{bpm}\right)$$

where T is the period of one complete cardiac cycle.^[^ ^108^ ^]^ However, a detailed explanation of the HR monitoring device's working mechanism is out of scope of this review. The details can be found elsewhere.^[^ ^109^, ^110^ ^]^

## 4 Design Considerations in Organic Oximetry

For generating quality PPG signal magnitudes with high SNR for accurate SpO_2_ measurements, the main critical parameters involved in the efficient design of organic PPG sensors involve the careful consideration of the geometries for OLED and OPD. The most common geometries proposed in the literature include arrays, circular geometry, rectangular geometry, and bracket geometry. This section gives a brief summary of the various geometry considerations for OLED and OPD and their effect on PPG signal magnitude is covered.

### 4.1 Arrays

**Figure 3:**
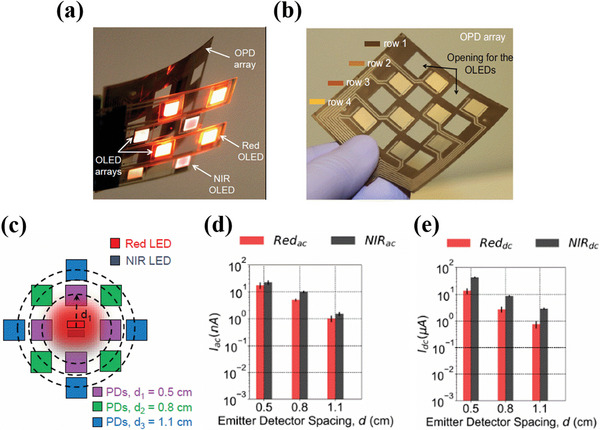
a) Pulse oximeter array assembly consisting of OLEDs and OPDs fabricated on two different substrates. b) OPD array consisting of 8 OPD pixels with two pixels in each row placed at an alternate position. Empty pixels for OLEDs at every adjacent position of OPD are also shown. c) Schematic used in the study to investigate the effect of OLED‐OPD spacing distance on the red and NIR PPG signal magnitude. d) and e) Images showing the effect of emitter spacing distance on the red and NIR AC and DC signal magnitudes with emitter detector spacing distances of 0.5, 0.8, and 1.1 cm respectively. Reproduced with permission.^[^ ^94^ ^]^ Copyright 2018, National Academy of Sciences.

Khan et al. demonstrated a pulse oximeter consisting of OLEDs and OPDs fabricated in the form of arrays on two different substrates as shown in **Figure** **3**a. The OPD array consisted of a 4×4 matrix design with OPDs placed at every alternate position arranged in 4 different rows for a total of 8 OPDs, as shown in Figure 3b. On the other hand, the OLED array consisted of alternate pixels of NIR and red‐emitting OLEDs arranged in 4 different rows. Altogether row 1 and 3 consisted of four red OLED pixels and row 2 and 4 consisted of four NIR OLED pixels. Both the OLED and OPD arrays were finally stacked one on top of the other to complete the array oximeter design consisting of NIR and red‐light emitting pixels at every alternate position relative to the OPD. The OLED‐OPD spacing distance was set to 0.5 cm while the active pixel and sensing area were 0.7  ×  0.7 cm^2^ each for OLEDs and OPDs respectively. The complete array was 4.3 cm both in length and width with a total of nine pulse oximeter pixels. To understand the effect of OLED‐OPD spacing distance (*d*) on the AC and DC signal magnitudes, a design was studied where four OPDs were positioned on three concentric circular rings at 0.5, 0.8, and 1.1 cm from NIR and red‐light source, as shown in Figure 3c. AC and DC PPG signal magnitudes were recorded while the sensor was positioned on the wrist. The highest SNR was obtained for an emitter‐detector spacing distance of 0.5 cm. An increase of a spacing distance (*d*) beyond 0.5 cm affected a decrease of both AC and DC signal magnitudes because of the signal losses due to scattering and long path length of the reflected light before reaching the detector as shown in Figure 3d and Figure 3e respectively. Further, reducing the distance between OLED and OPD of <0.5 cm, suffered direct coupling of light between emitter and detector resulting in saturated DC current. However, such a spacing distance (*d*) value could vary from design to design. Additionally, by knowing the minimum flux required for the emitter to resolve the PPG signal, the spacing distance (*d*) can be further adjusted. Besides the spacing distance (*d*), other factors affecting the signal magnitude include: active area of OLED and OPD, incident optical flux from the OLED, and quantum efficiency of the OPD.^[^ ^94^ ^]^

### 4.2 Circular Geometry

**Figure 4:**
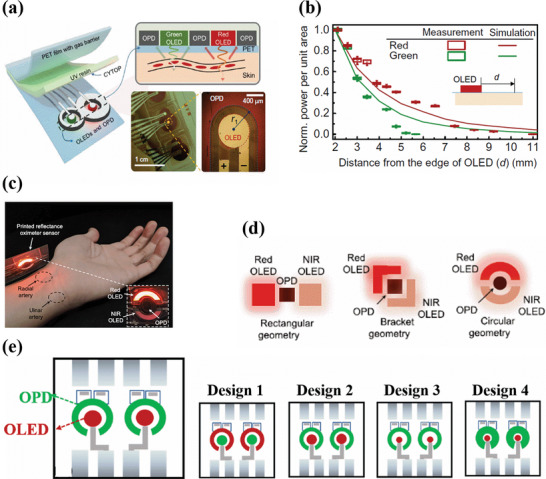
a) Schematic of the oximeter sensor with 8 shaped OPDs surrounding the green and red OLEDs. (b) Measured (indicated by box chart) and simulated (indicated by line chart) normalized power per unit area at different distances from the edge of the OLED. Reproduced with permission.^[^ ^81^ ^]^ Copyright 2018, American Association for the Advancement of Science. c) Pulse oximeter sensor with a circular design consisting of red and NIR OLED surrounding a central OPD. Sensing locations are marked for radial and ulnar arteries. d) Sensor geometries for ppg sensor consisting of rectangular geometry (left), bracket geometry (middle), and circular geometry (right). Reproduced with permission.^[^ ^111^ ^]^ Copyright 2019, IEEE. e) Represents the different circular designs with various combinations of OLED and OPD positions and sizes. Reproduced with permission.^[^ ^112^ ^]^ Copyright 2020, American Chemical Society.

An optimal design of oximetry organic sensors takes into account the careful consideration and understanding of the complexities involved in the propagation of light through the skin and various other organelles and multiple absorption and scattering effects. By adequate optical modelling and taking into account the above‐mentioned factors, Lee et al. derived a suitable design for PPG sensors. They fabricated a reflective‐based pulse oximetry system consisting of circular green and red OLEDs wrapped by 8‐shaped OPD, as shown in **Figure** **4**a. The radii of the red and green OLEDs were fixed to 0.4 mm each. Further, the OPD was designed to collect a maximum of the reflected light with high SNR after attenuation through the skin surface. In order to understand the spatial distribution of reflected light around green and red OLED, an optical skin model followed by Tuchin and co‐workers was chosen and the effective light propagation from OLED to OPD was simulated taking into account the reflection and scattering parameters through skin and organelles. Both experimental and simulated data showed that the spatial distribution of reflected light power densities in the lateral direction from red and green OLEDs follow a spatial decay with the distance from the edge of each OLED as shown in Figure 4b. Further in this design, the effect on SNR with different widths of the circular OPD was investigated. It was observed that for the incident light intensity of 0.01 W m^−2^ and OLED‐OPD spacing distance of 0.2 mm, the maximum SNR was observed for an effective OPD width of 2.8 mm for green light and 3.6 mm for red light.^[^ ^81^ ^]^ Increasing the OPD width beyond this optimum value followed a decay in the SNR.

In another circular design, Khan et al. fabricated a PPG sensor consisting of OLEDs in the shape of block arcs surrounding the circular OPD as shown in Figure 4c. In‐order to understand the role of sensor geometry on the PPG signal magnitude, three different geometries, including rectangular geometry (square‐shaped OLEDs are placed on either side of OPDs), bracket geometry (bracket‐shaped OLEDs are placed on either side of OPDs) and circular geometries (block arcs of OLEDs surrounding circular OPDs) were considered, as shown in Figure 4d. The OLED‐OPD spacing distance was kept at 2 mm for all three geometries. PPG signal magnitudes for the bracket and circular geometries were compared with the rectangular geometry. For the bracket geometry, a 39.7% improvement in the PPG signal magnitude with red light, and an 18.2% improvement with NIR light was observed. For the circular geometry, 48.6% and 9.2% improvements in the PPG signal magnitude were observed for the red light and NIR light respectively. Among all three geometries, the rectangular geometry showed the weakest signal, since the OPD is not completely surrounded by OLEDs and therefore is more sensitive to ambient light, hence generating more interferences and noise signals. Moreover, in the rectangular geometry, the light from the far edge of the OLEDs barely reaches the OPD, leading to increased optical losses and poor PPG signals. In addition, the effect of OLED‐OPD spacing distance on the PPG signal magnitude is also investigated. For each of the geometries, the PPG signal magnitude was recorded by varying the OLED‐OPD distance at 2, 4, and 6 mm, respectively. For all three sensor geometries, the magnitude was highest for a 2 mm distance.^[^ ^111^ ^]^ With these results, it appears clearly that circular geometry is more efficient than bracket or rectangular geometries.

Further, in a circular geometry, the OPD can be either at the center of the geometry with OLEDs surrounding it, or vice versa. Accordingly, Bilgaiyan et al. investigated the effect of size and position of circular red OLED and OPD on the PPG signal magnitude. In design 1, the OPD was placed at the center while the OLED was surrounding it. In design 2, the OLED was placed at the center while the OPD was surrounding it. Peak‐to‐peak PPG signal magnitudes of 90 mV and 100 mV were obtained for the geometries 1 and 2 respectively. However, despite reasonable PPG signal magnitudes, because of the large area of the ring OLED in design 1, the power consumption to drive it was three times higher than for design 2. Moreover, the lower PPG signal magnitude for design 1 may be due to the fact that some of the reflected light photons are lost, and not detected by the small circular OPD placed inside. Design 2 was therefore further investigated by reducing the area of the OLED and increasing the OLED/OPD spacing distance. However, in design 3 the peak‐to‐peak PPG signal magnitude was reduced by 30 mV, suggesting that the spacing distance was too large. Finally, in design 4, keeping the same OLED area as in design 3 and reducing the OLED/OPD spacing distance to an optimum value of 800 µm, the highest peak‐to‐peak PPG signal magnitude of 120 mV was obtained.^[^ ^112^ ^]^ The different circular designs are shown in Figure 4e.

## 5 Processing techniques, Materials, and Device performance

OLEDs and OPDs constitute the organic optoelectronic components of the organic pulse oximeter. As their performances will directly affect the sensor efficiency, it is of prime importance to carefully select sensor materials and follow efficient fabrication techniques. The fabrication of OLEDs and OPDs altogether on thin and flexible substrates with organic semiconductor materials will allow for the maximum process advantages in most favorable form factors. The fabricated OLED should provide the necessary light flux able to obtain a good PPG signal with high SNR (signal‐to‐noise ratio). On the other hand, an OPD operating under short circuit conditions (0 V bias) is able to sense low light signals due to reduced dark currents allowing for increased external quantum efficiency (EQE) resulting in improved PPG signal magnitudes. In addition to this, other critical components involved in the fabrication of OLEDs and OPDs include the choice of substrates, transparent electrode material, active and interlayer materials, electrode interconnections, and encapsulation.

Due to their advantages in flexibility, lightweight, and softness, polymeric substrates are generally chosen for flexible OLEDs and OPDs. Thin plastic films made from parylene,^[^ ^113^ ^]^ polyester (PET),^[^ ^114^ ^]^ polyimide (PI),^[^ ^115^ ^]^ and polyethylene napthalate (PEN),^[^ ^116^ ^]^ are good candidates as they exhibit excellent mechanical characteristics under bending. In addition, a flexible and transparent electrode is an essential component for the realization of flexible OPDs and OLEDs. Despite their brittle nature, indium titanium oxide (ITO) and indium zinc oxide (IZO) based transparent electrodes still dominate as the best choice for anode material in OLEDs and OPDs, due to their excellent sheet resistance (10–20 Ω Sq^−1^) and optical transmission properties (>80%).^[^ ^117^ ^]^ However, there are some alternate flexible materials with comparable optical and electrical properties as ITO, that could be promising candidates to replace it: conducting polymers,^[^ ^118^, ^119^ ^]^ metal nanowire networks,^[^ ^120^, ^121^ ^]^ carbon nanotubes,^[^ ^122^, ^123^ ^]^ graphene and composites of these materials.^[^ ^124^ ^]^ Electrode interconnections are also significant for efficient electrical contacts. A thin layer of gold is commonly used for interconnections, due to its flexible characteristics that are suitable for conformal and flexible devices.^[^ ^125^, ^126^ ^]^ Other possibilities include conducting polymers and liquid metal‐based conductors.^[^ ^127^ ^]^

Alongside, a low turn‐on voltage, 3 V or less is typically required for an efficient OLED.^[^ ^128^, ^129^, ^130^, ^131^ ^]^ Additionally, low operating voltages reduce the power consumption of the OLED leading to improved energy efficiency. These factors we believe are very crucial in wearable devices such as pulse oximeters as they depend on finite energy sources such as batteries for power sources. Furthermore, the low turn‐on voltage of OLEDs is affected by several factors including the choice of organic materials, energy level alignment, doping, carrier mobility, contact resistances, injection/ blocking interlayers, electrode materials, structure and configuration of the OLED layers stacking and device engineering.^[^ ^132^, ^133^, ^134^, ^135^, ^136^, ^137^ ^]^ Further, active and inter‐layer materials for OLEDs and OPD consist of either solution‐processed polymers or vacuum‐evaporated small molecules. These materials when solution‐coated or vacuum coated under inert conditions will exhibit high charge carrier mobility due to minimum defect states and traps and improves efficiency.^[^ ^138^, ^139^, ^140^, ^141^ ^]^ Moreover, interlayers will enhance charge extraction and collection efficiency at the electrode interface.^[^ ^142^, ^143^, ^144^, ^145^, ^146^ ^]^ Solution processing techniques include screen printing, blade coating, and spin coating techniques.^[^ ^147^, ^148^ ^]^ This section is divided based on the processing technique involved in the fabrication of OLEDs and OPDs for an organic pulse oximeter and includes detailed information regarding the choice of structure, materials, and device performance in comparison with the commerciallyavailable inorganic pulse oximeter sensors.

### 5.1 Solution Processing

**Figure 5:**
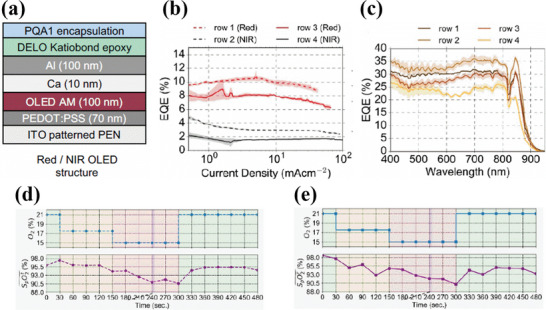
a) Device structure of the red/NIR OLED. b) EQE of the NIR and red OLEDs from rows 1, 2 (dotted lines) and 3, 4 (straight lines). c) EQE of the OPDs in different rows marked with distinct colors. The average EQE is ≈30% throughout the absorption spectrum. d) SpO_2_ measurements from the commercial pulse oximeter where the oxygen concentration is varied from 21% to 15% and back to 21%. e) SpO_2_ measurements of the organic reflection probe attached to the fore head. Reproduced with permission.^[^ ^94^ ^]^ Copyright 2018, National Academy of Sciences.

Khan et al. fabricated a reflective flexible pulse oximeter sensor array consisting of red and NIR OLEDs and OPDs. Both the OLEDs and OPDs were fabricated on two different PEN substrates and assembled together (design and assembly are explained in Section 4.1 of the review). The OLED and OPD arrays were fabricated using the standard structure and had the same stack, except for the different active layer materials. They both included patterned ITO as an anode and blade‐coated PEDOT: PSS as a hole transport layer as patterning the ITO reduces an otherwise large parasitic capacitance between the PEDOT: PSS layer and the remaining stack of the sensor. The active material for OPD consisted of 1:2 mixture of cyclopentadithiophene (CDT) based donor and PC71BM acceptor. The emissive and active layers for the OLED and OPD were blade coated, followed by thermally evaporated Ca/Al and Al as a cathode contact for the OLED and OPD respectively. Silver interconnections were screen printed between the anode and cathode of the OLED and OPD and the external circuit. Every OLED pixel was encapsulated by UV‐curable epoxy and a plastic film. The structure of the fabricated OLED is shown in **Figure** **5**a. The red and NIR OLEDs had a peak emission at 612 and 725 nm respectively. The turn‐on voltage for the NIR and red OLED was 3 V. For oximetry, the OLEDs were operated at a current density of 10 mA cm^−2^, giving an optical flux of 0.9 and 0.2 mW for red and NIR light respectively. At these operating conditions, the EQE of the OLEDs was in the range of 8–10% for red OLED and 2–3% for NIR OLED respectively (Figure 5b). Moreover, the less optical flux requirement for NIR OLED is associated with the fact that the NIR light can penetrate deep inside the tissue without significant loss in light scattering due to its larger wavelength. The fabricated OPD showed an average EQE value of 30% throughout the absorption spectrum, as shown in Figure 5c. The cut‐off frequency of the OPD at −3 dB was over 5 kHz, which is 5 times higher than the sufficient range for pulse oximetry. For the comparative test, the organic sensor was placed on the forehead of an individual, and reflection mode measurements were compared with the transmission mode measurements of a commercial probe attached to a finger. An altitude simulator was used to vary the oxygen concentration in the air inhaled by the individual through a face mask. The change in oxygen concentration caused the SpO_2_ in the individual's blood to vary. During the test, the inhaled oxygen concentration varied from 21% to 15% and back to 21% in a time interval of 8 min. In the first 30 s of the experiment, a base line of 21% oxygen concentration was maintained. During this time interval, the commercial finger probe showed a SpO_2_ of 96% (Figure 5d) and the fabricated array sensor showed a SpO_2_ of 98% (Figure 5e). Between *t* = 30 s and 150s, the oxygen concentration was reduced and maintained at 17.5%. After this time, the oxygen concentration was further reduced to 15% and kept at the same percentage until *t* = 300 s when the SpO_2_ values of the commercial probe and the array sensor were 90.5% and 90.4% respectively Finally, at *t* = 300 s the oxygen concentration was increased back to 21%, and the SpO_2_ values of the commercial probe and the array sensor were respectively 94.5% and 93.5%. A mean error of 1.1% is found between the organic oximeter sensor and the commercial sensor. However, when the experiment is performed on the finger instead of the forehead and compared with a commercial oximeter, the mean error is only 0.48%, thus providing a comparable device performance. However, the difference in the mean error is due to the different pulse arrival times that create a measurement delay during the comparison measurements between the organic pulse oximeter probe on the forehead and the commercial pulse oximeter probe on the finger.^[^ ^94^ ^]^

**Figure 6:**
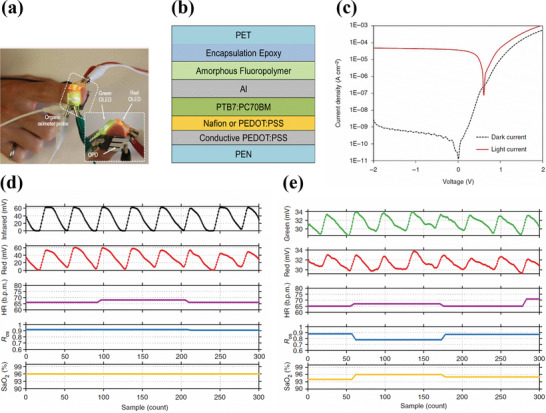
a) Schematic of an oximeter sensor in transmission mode with green and red OLEDs placed on top of a finger and OPD on the opposite side. b) Structure of the OPD. c) Dark current (black dotted line) at ‐2 V and photocurrent (red solid line) under the illumination of 355 µW cm^−2^ of 640 nm red light. d,e) Simultaneous SpO_2_ measurements on the finger from commercial oximeter and organic oximeter respectively. The heart rate recorded was in the range of 60–70 beats per minute for both commercial and organic oximeters (magenta‐colored line). The R_os_ is shown in blue lines for both in‐organic and organic oximeters. Reproduced with permission.^[^ ^98^ ^]^ Copyright 2014, Springer Nature.

In another approach, Lochner et al. fabricated a transmission mode pulse oximeter consisting of green and red polymer‐based LEDs (PLEDs) and an OPD as shown in **Figure** **6**a. The fabricated PLEDs had a peak emission of 532 and 626 nm for green light and red light respectively. Under short circuit conditions, the OPD exhibited improved EQE up to 38% for green light and 47% for red light due to the incorporation of a stable and efficient PTB7:PC71BM‐based donor‐acceptor bulk heterojunction system. Moreover, even at ‐2 V, the OPD showed dark current densities as low as 1 nA cm^−2^ indicating low leakage currents. Both OLEDs and OPD were fabricated in the standard structure with ITO as bottom transparent anode and thermally evaporated LiF/Al as cathode. The emissive layer of the green PLED was spin‐coated from a blend of polyfluorene derivatives consisting of poly(9,9‐dioctylfluorene‐co‐n‐(4‐butylphenyl)‐diphenylamine) (TFB) and poly((9,9‐dioctylfluorene‐2,7‐diyl)‐alt‐(2,1,3‐benzothiadiazole‐4,8‐diyl)) (F8BT) in the ratio 1:9, whereas the emissive layer of the red PLED was spin‐coated from a tri‐blend constituting, TFB, F8BT and poly((9,9‐dioctylfluorene‐2,7‐diyl)‐alt‐(4,7‐bis(3‐hexylthiophene‐5‐yl)−2,1,3‐benzothiadiazole)−20,20‐diyl) (TBT) in the ratio 25:70:5 respectively. Both PLEDs had the same active area of 4mm^2^. On the other hand, the transparent electrodes and active layer for the OPD were blade coated on the flexible PEN substrate. The devices were finally encapsulated by UV‐curable epoxy. The structure is shown in Figure 6b. The irradiance of the green and red PLED was 20.1 and 5.83 mW cm^−2^ respectively at an operating voltage of 9 V for oximetry measurements. Owing to its shorter wavelength usually green light suffers penetration depth thereby giving low PPG signals magnitudes. Hence typically, a higher flux of green light is usually employed in pulse oximeters when compared with red light.^[^ ^149^ ^]^ Furthur, the OPD in this study was able to detect a dark current as low as 1 nA cm^−2^ at −2 V bias. The photocurrent and dark current versus voltage plot of the OPD under the illumination of 355 µW cm^−2^ of red light at −2 V bias is shown in Figure 6c. During the measurements, in order to sense very low photocurrents, the OPD was biased at 0 V. The cut‐off frequency of the device at ‐3 dB was higher than 10 kHz. The SpO_2_ values obtained from the commercial oximeter (Figure 6d) and with the organic oximeter sensor (Figure 6e) during simultaneous measurements showed similar oxygen saturation values in the range of 94–96% with a 2% error, thus indicating similarities in device performances. In addition to this, the performances of PLED and OPD in the PPG signal generation are evaluated in combination with the commercially available Si‐based photodiodes or LEDs. The PPG signal magnitudes recorded from the pulse oximeter in combination with OLEDs and Si photodiode showed a PPG signal magnitude of 16 mvp‐p for green light and 6 mvp‐p for red light, whereas the PPG signal magnitudes obtained with a combination of OPD and inorganic LEDs showed values of 26 mvp‐p for green light and 16 mvp‐p for red light. The decrease in PPG signal magnitude in the former is due to the low optical power of the PLEDs. The higher PPG signal magnitude in the latter case is due to the incorporation of inorganic LEDs with higher optical power. However, the PPG signal magnitudes recorded with the combination of OPD and PLEDs showed only 3 mvp‐p for green light and 2.5 mvp‐p for red light. The low magnitude of the PPG signals is associated with low photocurrent from the OPD and low optical flux from the PLED. In order to match the PPG signal magnitudes with their inorganic counterpart, the active area of the OPD was increased to 21mm^2^ to sense higher photocurrents with a larger active area that could generate higher and comparable magnitude PPG signals.^[^ ^98^ ^]^

**Figure 7:**
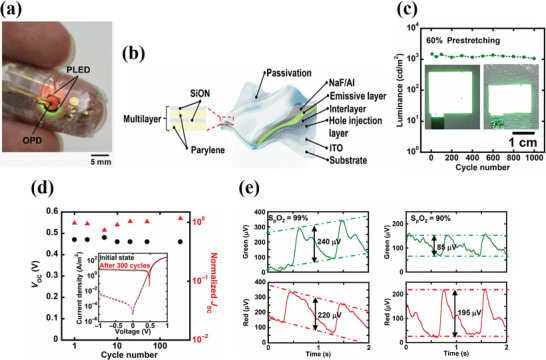
a) Image of an ultra‐flexible pulse oximeter sensor attached to a finger. b) Structure of an ultra‐flexible PLED whose encapsulation consists of alternating inorganic (200‐nm‐thick SiON) and organic (500‐nm‐thick parylene) layers. c) Cyclic stretching test of the green PLED. The inset is the image of the PLED before and after the mechanical test. d) The plot of *V*_oc_ versus normalized *J*_sc_ with the number of stretching cycles for the OPD. The black circles and red triangles represent the *V*_oc_ and normalized *J*_sc_ respectively. The inset figure shows the JV characteristics of the OPD before and after stretchability tests. e) PPG signal magnitudes for red and green light in normal conditions with 99% oxygen saturation (left) and 90% in the reduced oxygen saturation condition (right). Reproduced with permission.^[^ ^150^ ^]^ Copyright 2016, American Association for the Advancement of Science.

A similar approach of fabricating organic PPG sensors using green and red light but working in the transmission mode is demonstrated by Yokota et al. (**Figure** **7**a). The fabricated smart electronic sensory system consisted of green and red PLEDs and OPD. The dimensions of the active area of both green and red PLED were 2×2 mm^2^. Both PLEDs were fabricated following the standard structure on a 1 µm thin parylene substrate. A 500 nm polyimide layer was coated on top of the parylene as a planarization layer. As a result, the surface roughness of parylene was reduced from 2.9 nm to 0.25 nm. A thin 70 nm of sputtered ITO was used as anode for the PLED stack. Further, the active layer consisted of spin‐coated phosphorescent‐based emissive material. Finally, NaF/Al was deposited as cathode. The PLEDs were encapsulated with alternating layers of inorganic (200 nm thick SiON) and inorganic (500 nm thick parylene) materials respectively. The structure of the PLED along with passivation layers is shown in Figure 7b. The total thickness of the PLED device was only 3 µm. On the other hand, the OPD was fabricated following an inverted structure on a 1 µm parylene substrate. Polyethylenimine ethoxylated (PEIE) acted as a hole‐blocking layer between ITO and P3HT: PCBM active layer of the OPD. Both the interlayer and active layer were spin‐coated. Finally, MoO_3_/Au was deposited as anode, followed by 1 µm parylene as a passivation layer. On the other hand, the green and red PLED had a peak emission wavelength value of 517 and 609 nm, respectively. Further, at a current density of 10 mA cm^−2^, the green and red PLEDs exhibited EQE of 13.9% and 12.4% and a luminance value of 4900 and 2100 cd m^−22^, respectively. In addition to the investigation of the optoelectrical properties of OPD and PLEDs, the mechanical properties of the performance of the PLEDs and OPDs under compressional stress are evaluated. Stretchability measurements were recorded for the PLEDs during mechanical testing. With a pre‐stretch value of 60%, the green PLED was subjected to 1000 stretching cycles. At the end of the test, the PLED showed only a 10% decrease in irradiation intensity (Figure 7c). On the other hand, the OPD was subjected to compressional stress from 0 to 40%. During the entire 300 stretching cycles, the variations of OPD's short circuit current density (*J*_sc_) and open‐circuit voltage (*V*_oc_) remained relatively small (Figure 7d). These results ensured the superior mechanical properties of PLEDs and OPD and their suitability for conformal PPG sensors. For the pulse oximeter measurements, the structure consisting of red and green PLEDs and OPD was applied on a finger using soft elastomeric adhesive tape. The driving voltage of the PLEDs was set at 5 V at a switching frequency of 5 s. Percentage oxygen saturation at normal and low oxygen concentrations were evaluated by recording the red and green PPG signal magnitudes. The values showed 99% and 90% under normal and reduced oxygen concentration respectively (Figure 7e), thus indicating promising results.

### 5.2 Vacuum Processing

**Figure 8:**
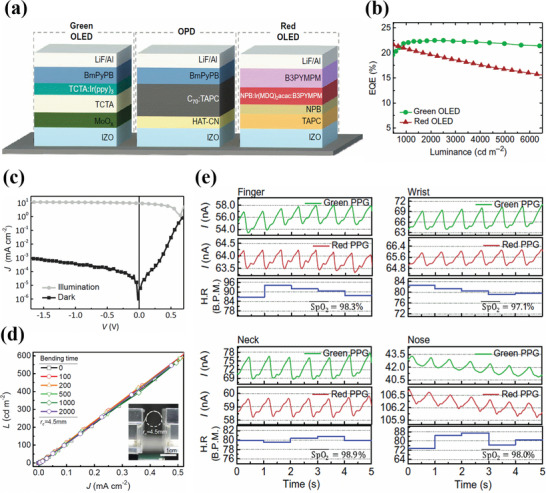
a) Structure of green OLED, red OLED, and OPD. b) EQE of the green and red OLEDs as a function of luminance. c) J–V characteristics of the OPD under dark and illumination (AM 1.5). d) Luminance as a function of current density for the OLED after several bending cycles. The inset is the experimental set up for the bending test. e) PPG signal magnitudes for red and green light illumination along with the heart rate and SpO_2_ values at different sensing locations such as finger (left top), wrist (right‐top), neck (left bottom), and nose (right bottom). Reproduced with permission.^[^ ^81^ ^]^ Copyright 2018, American Association for the Advancement of Science.

Seunghyup et al. designed a reflective mode circular PPG sensor consisting of green and red OLEDs operating with low turn‐on voltages of 2.1 and 2.8 V and high EQE of 20.8% and 21.9% at a luminance of 1000 cd m^−2^ for red and green OLEDs respectively. The EQE characteristics of the OLEDs are shown in **Figure** **8**b. The low turn‐on voltages and high EQE for the OLEDs are associated with the incorporation of tris[2‐phenylpyridinato‐C2,*N*]iridium(III) (Ir(Ppy3)) and tris[2‐phenylpyridinato‐C2,*N*]iridium(III) and bis(2methyldibenzo[f,h]quinoxaline) (acetylacetonate)iridium(III) (Ir(MDQ)2acac) as efficient iridium based phosphorescent light emitting materials for green and red OLEDs respectively. (the design of the sensor is illustrated in Section 4.2 of the review). Further, the OLEDs and OPD were fabricated on a PET substrate in the standard structure with RF‐sputtered indium zinc oxide (IZO) as the anode (Figure 8a). The green OLED consisted of the stack of PET/IZO/MoO_3_/TCTA/TCTA:Ir(Ppy)_3_[8 wt.%)]/BmPyPB/LiF/Al and the red OLED stack was PET/IZO/TAPC/NPB/NPB:B3PYMPM:Ir(MDQ)2acac(9 wt.%)/B3PYMPM/LIF/Al. On the other hand, the OPD stack consisted of PET/IZO/HAT‐CN/C70: TAPC (4 wt.%)/BmPyPB/LiF/Al. The active layer of the OPD was thermally evaporated from a blend of C70 and TAPC in the ratio 20:1. The device was finally encapsulated with CYTOP (fluorinated polymer) followed by spin coating of a UV curable resin over the sensor. The green and red OLEDs had a peak emission wavelength of 520 and 610 nm, respectively. During the device operation, the irradiance of the OLEDs was set at 50µW cm^−2^ with an irradiation area of 5 × 5 cm^2^ and a spatial variation of 2.5%. The low irradiance of the OLEDs is associated with the high responsivity of the OPDs which was 0.29 A W^−1^ for red light and 0.21A W^−1^ for green light. Moreover, the green OLED was driven at 5 V at a driving current of 25 µA and the red OLED was driven at 3.3 V at a driving current of 21 µA. The dark current at 0 V was in the range of a few nanoamperes, as shown in Figure 8c. The shot noise limited detectivity of the OPD was 3.2 × 10^10^ and 2.3 × 10^10^ Jones respectively for the green and red light. Further, the flexibility of the OLEDs was checked by subjecting them to several bending tests at a fixed radius of curvature (*r*_c_ = 4.5 mm) and the luminance was recorded at different current densities. The luminance characteristics showed a similar trend after several bending cycles, as shown in Figure 8d. The SpO_2_ measurements in the reflection mode were recorded at the different sensing locations such as finger, wrist, neck, and nose and showed similar values of 98.3%, 97.1%, 98.9%, and 98% respectively (Figure 8e),^[^ ^81^ ^]^ thus indicating good efficiency and accuracy of the organic PPG sensor.

## 6 Encapsulation

**Figure 9:**
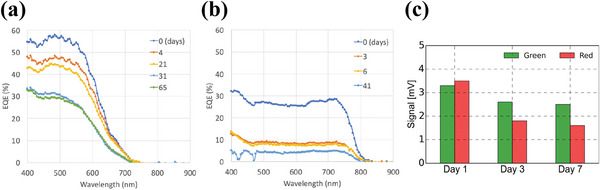
Degradation of EQE performance of the OPD in air. a) The OPD is based on a PCDTBT:PCBM70 active layer b) The OPD is based on a P3HT:O‐IDTBR active layer. Reproduced with permission.^[^ ^95^ ^]^ Copyright 2020, Wiley‐VCH. c) Decrease in PPG signal magnitude along with the number of days. Reproduced with permission.^[^ ^98^ ^]^ Copyright 2014, Springer Nature.

Flexible organic pulse oximeter sensors with extended ambient stability in air would allow for long‐term realistic and unobstructed bio‐integration. One of the largest limitations for practical and extended operation is the stability of organic devices, since organic semiconductor active layers degrade with time due to the reactions with atmospheric air and oxygen, resulting in poor performances.^[^ ^151^, ^152^ ^]^ For example, in the pulse oximeter sensor fabricated by Han et al., the OPD made with a PCDTBT: PCBM70 active organic layer, showed an EQE value above 50% of the initial EQE peak, for a period of 20 days (**Figure** **9**a). On the other hand, the EQE of the OPD based on P3HT: O‐IDTBR active layer dropped below 50% of the initial EQE peak value, just after 3 days of its fabrication (Figure 9b).^[^ ^95^ ^]^

Typically, a water vapor transmission rate (WVTR) of 10^−6^ g cm^−2^ per day for OLEDs and 10^−3^ to 10^−4^ g cm^−2^ per day for OPDs is essential to meet the long lifetime stability for practical device applications.^[^ ^150^ ^]^ Moreover, for ultra‐flexible and conformal applications, substrates with thicknesses of a few micrometers or less are easily deformed because of thermal expansion during high‐temperature processing such as plasma deposition, and hence are susceptible to damage.^[^ ^153^ ^]^ Therefore, the passivation on such thin substrates has to be done in low‐temperature processing conditions and must comply with low layer thicknesses in order to maintain intrinsic flexibility. Some of the most common low‐temperature encapsulation processes for organic devices include transparent flexible barriers, epoxy adhesives, and plastic substrates.^[^ ^154^ ^]^ However, these materials offer lower protection from ambient oxygen and moisture and thus are not suitable for long time stability. For example, in the PPG sensor array fabricated by Khan et al., a DELOs UV curable adhesive followed by a plastic film (PQA1) was used.^[^ ^94^ ^]^ In the PPG sensor fabricated by Lochner et al., a DELOs UV curable adhesive was also used. The PPG signal magnitudes for green and red light recorded on day 1 after encapsulation showed a peak‐to‐peak value of 3.3 mV for green light and 3.5 mV for red light. However, on day 3 the PPG signal magnitudes reduced to 2.6 mV for green light and 1.8 mV for red light. The PPG signal magnitudes on day 7 were further reduced to 2.5 mV for green light and 1.6 mV for red light. In Figure 9c, a bar graph depicts the decrease in PPG signal magnitude for green and red light with the days count. The origin of this decrease comes primarily from the poor encapsulation of the optoelectronic sensor.^[^ ^98^ ^]^ On the other hand, multilayer thin‐film encapsulation consisting of organic/inorganic materials is gaining significance in recent years due to its superior moisture and oxygen barrier properties, by combining the advantages of inorganic and organic materials. In multi‐layered encapsulation, the inorganic layer contributes to preventing oxygen and moisture penetration, whereas the organic layer decouples the pinhole and surface defects of the inorganic layer, thereby improving the effectiveness of the encapsulation.^[^ ^155^ ^]^ For example, the PPG sensor fabricated by Takao Someya et al. consisted of passivation layers with alternate 200 nm inorganic (SiON) and 500 nm organic (Parylene) materials. Coating 5 alternate SiON and Parylene layers helped to achieve a WVTR of 5×10^−4^ g cm^−2^ per day. With the incorporation of the passivation layers, the luminance‐dependent half‐life of the OLED increased from 2 to 29 hours at 20 °C and 60% relative humidity, at a current density of 12.5 mA cm^−2^. After encapsulation, the device showed a constant PPG signal magnitude, for up to four days when operated in air.^[^ ^150^ ^]^

## 7 2D Oxygen Mapping Using Pulse Oximetry

**Figure 10:**
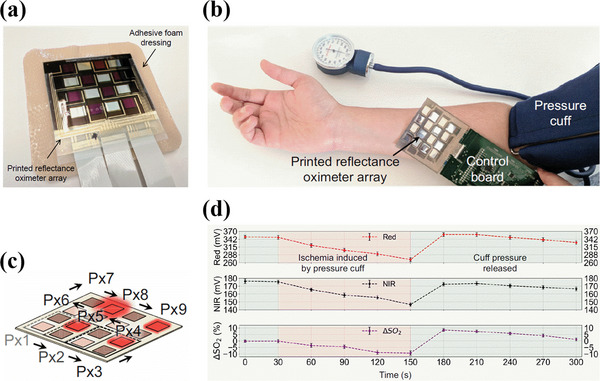
a) Pulse oximeter sensor array with adhesive foam dressing. b) Printed reflectance oximeter array on an individual forearm connected to control electronics. The supply of blood is controlled by a temporary pressure cut‐off. c) Schematic showing the scan direction from the pixel Px1 to Px9 during operation. d) ΔSpO_2_ values along with measured red and NIR PPG signal magnitudes as a function of time, shown in purple, red and black dotted lines respectively. Each dot represents the mean value obtained from 9 pixels and the error bars represent the standard deviation of the data. Time intervals for ischemia condition and pressure cut‐off are highlighted. Reproduced with permission.^[^ ^94^ ^]^ Copyright 2018, National Academy of Sciences.

Conventional pulse oximeters make use of single‐point area measurements, either in the reflection or transmission mode, to determine the SpO_2_ values. They intrinsically have a limitation for large‐area SpO_2_ measurements. In extreme medical conditions, like ischemia, where there is low blood perfusion, the limited supply of blood flow and oxygen to the tissues will generate a weak and non‐steady pulsatile PPG signal. Hence, in such a condition, either reflection or transmission mode oximetry at one point location cannot be performed. However, in such a case, the change in oxygen saturation (Δ*SpO*_2_) over a large area can be measured using the reflectance mode oximetry, due to time‐varying light attenuation. This way, the reflectance mode oximeter fabricated by Khan et al. could monitor the change in oxygen saturation during an ischemia condition (the design of the sensor consisting of printed red and NIR OLEDs and OPDs in the form of arrays is illustrated in Section 4.1. of the review). For the experiment, the sensor is placed on an individual arm, and for the conformal contact, the mounting of the sensor is done using adhesive foam dressing as shown in **Figure** **10**a. The pulse oximeter array consisted of OLEDs and OPDs arranged in 4 different rows and columns and consists of a total of 9 pixels (Px1 to Px9). Each pixel consists of two OPDs and two different OLEDs as shown in Figure 10c. In the experimental setup, the temporary ischemia condition was realized by applying a temporary pressure cut‐off (50 mmHg over systolic pressure) on an individual's arm, as shown in Figure 10b. During the operation, a raster scan was performed from Px1 to Px9. Data was collected from each pixel at a frequency of 500 Hz and was averaged to generate the 2D contour maps corresponding to the ischemia condition as shown in Figure 10d. The experiment clearly demonstrated the ability to detect a change in oxygen saturation on a large area during the ischemia condition.^[^ ^94^ ^]^

## 8 Factors Affecting the Sensor Performance

PPG signals are mainly affected by the sensor location, optical losses due to refractive index mismatch at the interface between the device and the skin, temperature effects, direct light coupling between OLEDs and OPDs, motion artifacts and pressure disturbances, effect of skin pigmentation, presence of dyshemoglobins and low blood oxygen levels. This section includes a brief description of the various factors mentioned above and their effect on the PPG signal magnitudes.

### 8.1 Sensor Location

**Figure 11:**
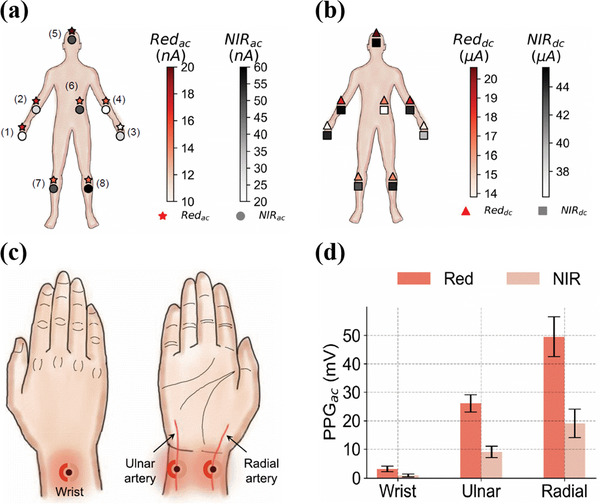
a) Sensor placement locations on the body indexed from 1–8. The fill color of the markers (★ for red and ○ for NIR) indicates the magnitude of the AC signal current at each location. The largest peak‐to‐peak AC signal current is recorded on the forehead while the lowest is recorded on the wrist. b) DC signal current recorded at the different sensing locations as marked in image 11a. The fill color of the markers (Δ for red and ◻ for NIR) indicate the magnitude of the DC signals. Reproduced with permission.^[^ ^94^ ^]^ Copy right 2018, Proceedings of the National Academy of Sciences. c) Sensing locations for the PPG signal measurements on top of the wrist, on top of the ulnar artery, and on top of the radial artery. d) AC PPG signals magnitudes from the wrist, radial artery, and ulnar artery. The error bars represent a set of 3 measurements on each sensing location. Reproduced with permission.^[^ ^111^ ^]^ Copyright 2019, IEEE.

Accurate SpO_2_ measurements require large SNR, and thus large AC and low DC PPG signal magnitudes. The AC and DC signal magnitudes recorded by the PPG sensor at a given set of operating conditions could vary at different sensing locations, mainly due to different thicknesses of epidermal tissues surrounding the blood vessels. Moreover, discrepancy in PPG signals magnitudes is more likely to occur when the PPG signals are recorded simultaneously at different sensing locations, due to the different pulse arrival times. The commonly used sensing location for the PPG sensor is fingers, as they have high SNR and also can be used either in reflection or transmission mode oximetry. However, this sensing location hinders continuous and real‐time monitoring in practical applications, as the fingers are used for multitasking in our daily physical activities. Several alternative measurement locations are investigated that include earlobes, wrist, forehead, ribcage, etc. Khan et al. investigated the effect of sensing location on the AC and DC PPG signal magnitude in the reflection mode, by placing the organic PPG sensors at 8 different locations on the human body, as shown in **Figure** **11**a. The largest AC and DC PPG signal magnitudes were recorded on the forehead, with a maximum AC signal current of 20 nA for red light and 60 nA for NIR light, making it the best suitable location for oximetry. Moreover, the forehead area is less prone to movement and therefore has the highest SNR. The lowest AC signal intensity was recorded on the wrist, with values nearly half of those recorded on the forehead. DC PPG signal magnitudes measured on the ribcage showed the lowest signal magnitude for NIR light, as shown in Figure 11b.^[^ ^94^ ^]^ In another study, Lee et al. measured the AC to DC signal ratios with organic PPG sensors placed on wrist, neck, and fingertip for the red light operating at 610 nm. The largest AC to DC signal ratio was recorded on the wrist with a value of 0.20, while on the fingertip and neck, the ratio value was 0.014 and 0.004 respectively. Furthur the LED current required for the PPG signal corresponding to an SNR >30 dB was recorded. The values recorded were 480, 240, and 240 µA for the neck, wrist, and fingertip respectively, indicating that the sensor located on the neck required twice as much as LED current to achieve a PPG signal of SNR >30 dB when compared with that on the wrist and fingertip. The low AC to DC signal ratios for the neck is associated with the different thickness of the epidermis surrounding the neck blood vessels.^[^ ^156^ ^]^ With another pulse oximeter sensor fabricated by Khan et al. the PPG signal magnitudes were recorded on the wrist, ulnar artery, and radial artery, and compared (Figure 11c). The experiments showed clearly a significant dependence of the PPG signal magnitudes on the sensing location. The weakest PPG signal was recorded for the wrist with a magnitude of 3.24 mV for red light and 0.94 mV for NIR light. The largest PPG signal was recorded for the radial artery with a signal intensity of 49.50 mV for red light and 19.08 mV for NIR light. On the ulnar artery, the PPG signal magnitude was 26.12 mV for red light and 9.02 mV for NIR light, as shown in Figure 11d. The weakest signal from the wrist was amplified 10 times for better resolution.^[^ ^111^ ^]^ In addition, the optimum sensing location further depends on the health condition of the patient. For example, in hypotensive patients, sensing at the earlobes or forehead is more reliable due to less vasoconstriction than at the fingertips.^[^ ^157^, ^158^ ^]^

### 8.2 Refractive Index Mismatch Between Substrate and Skin

**Figure 12:**
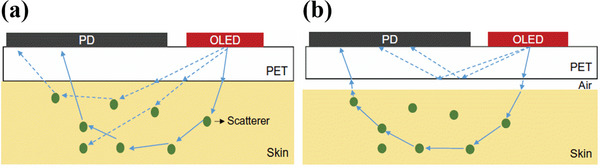
Schematic of the cross‐sectional view of the optical ray diagrams consisting of OLED and OPD. a) Substrate (PET) is optically coupled to the skin. b) Substrate with non‐conformal contact is not optically coupled with the skin, as it creates an air gap between the skin and the substrate. Reproduced with permission.^[^ ^81^ ^]^ Copyright 2018, American Association for the Advancement of Science.

Refractive index mismatch between the skin and substrate interface is an important factor that decides the quality of the PPG signals. About 30–35% of the total photons emitted by an OLED at the active layer undergo total internal reflection within the substrate.^[^ ^81^ ^]^ The loss factor is larger when the light couples with an air gap between substrate and skin, in non‐conformal contact as shown in **Figure** **12**b. When the refractive index of skin and substrate are similar, all photons can theoretically enter the skin with minimum optical loss, as shown in Figure 12a. Lee et al. monitored the light coming out from the edge of the substrate of bottom emission OLEDs when the bottom surface was in conformal contact with the skin. The optical loss was reduced by 32% when compared with the optical loss recorded in the non‐conformal contact mode. With the use of index‐matching fluid between the substrate and the skin, the optical loss was further reduced by 40%, which means there was a 10% improvement in optical coupling with the use of refractive index‐matching fluids.^[^ ^81^ ^]^

### 8.3 Temperature Effect

**Figure 13:**
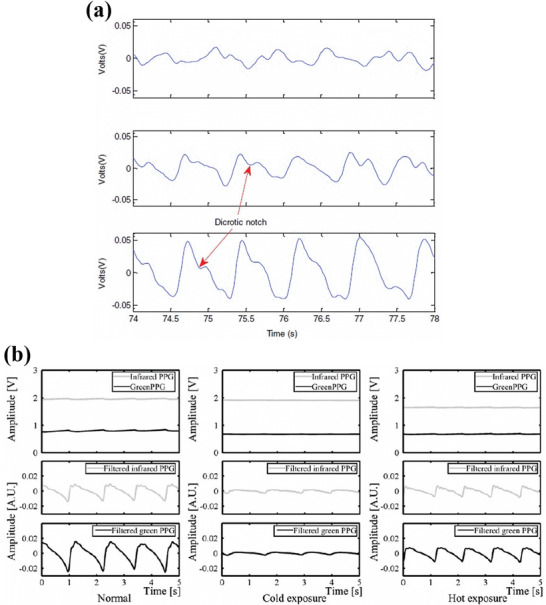
a) AC component of the PPG signal magnitude for NIR light during cold exposure condition (top panel), normal condition (middle panel), and warm exposure condition (bottom panel). The dicrotic notch positions marked in the image indicate the closure of the heart aortic valve during the cardiac cycle. Reproduced with permission.^[^ ^159^ ^]^ Copyright 2015, Elsevier Ltd. b) PPG signal magnitudes for green and NIR light during normal exposure (left), cold exposure (middle), and hot exposure condition (right). The top panels represent the DC component of the PPG signal magnitudes. The middle and bottom panels represent the bandpass filtered (0.7–10 Hz) AC components of the PPG signal magnitude for respectively IR and green light. Reproduced with permission.^[^ ^100^ ^]^ Copyright 2011, Springer.

Blood perfusion of the capillaries varies with the skin temperature which in turn alters the AC and DC signal strength of the PPG signal. Khan et al. studied the effect of local skin and ambient temperature conditions on the AC portion of the PPG signal, arising from the attenuation of infra‐red light (849 nm) in the transmission mode oximetry. The AC component of the PPG signal data of twenty individual subjects aged between 21 and 35 years were studied at cold (<22 °C), normal and warm temperatures (>30 °C). The median of the PPG signal values for these twenty individuals at cold, baseline, and warm temperatures were 0.0066, 0.0174, and 0.0289 V respectively, indicating that cold temperatures significantly hindered the PPG signal magnitude by a factor of 4 compared to warm temperatures. **Figure** **13**a shows the AC portion of NIR PPG signals recorded at cold, normal, and warm temperature conditions for an individual under test. The improved PPG signal quality at warm temperatures results from an increase in blood perfusion, due to increased arterial‐venous shunt openings, thereby improving systemic blood flow.^[^ ^159^ ^]^ Maeda et al. made a similar study to understand the effect of temperature on the AC and DC PPG signal magnitudes of 20 individuals aged 23.6±1.5 years, in a reflection mode oximetry consisting of green light (525 nm) and in a transmission mode oximetry consisting of NIR light (880 nm), both measurements performed on the index finger. The temperature of the skin was varied by dipping the hand in an isothermal bath for a certain fixed time interval. The experiment was performed at cold (≈20 °C), normal, and warm (≈38 °C) temperatures (Figure 13b).^[^ ^100^ ^]^ It appears clearly that the lowest AC signal magnitude was recorded at cold temperature conditions, and the largest AC and DC signal magnitudes were recorded at normal temperature, giving good quality PPG signals.

### 8.4 Direct Coupling of Light Between OLED and OPD

**Figure 14:**
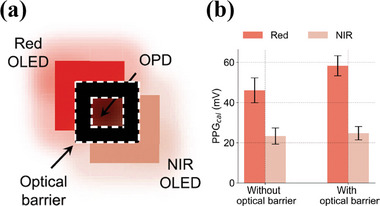
a) Schematic of a reflection‐mode pulse oximetry sensor in a bracket design providing an optical barrier between red/NIR OLEDs and a central OPD. b) The bar graph represents the PPG signals from the red and NIR channel with and without the optical barrier. The error bars represent the measurement variations coming from three different trials. Reproduced with permission.^[^ ^111^ ^]^ Copyright 2019, IEEE.

In the reflective sensing mode of a pulse‐oximeter, the light reflected through the skin carries the biological information, whereas the light scattered from the skin surface holds only some noise. The reflective mode oximetry is therefore somewhat susceptible to direct light coupling from the OLED to the OPD, especially when they are placed close to each other. The transmission mode oximetry is less affected by this phenomenon, since OLEDs and OPDs are placed opposite to each other. However, in reflective mode oximetry, optical barriers can be placed between OLEDs and OPD, thereby masking the scattered light, avoiding direct light, reducing the noise, and improving the SNR. As shown in **Figure** **14**a, Khan et al. introduced a bracket design made of an optical barrier in the form of black tape, placed between red and NIR OLEDs and an OPD. Consequently, a 26.5% improvement in the red‐light PPG signal was recorded. The PPG signal magnitudes for red and NIR light with and without optical barriers are shown in Figure 14b. It appears that the incorporation of the optical barrier doesn't affect the NIR PPG signal magnitudes, indicating that red light is more scattered on the skin surface than NIR light and is better blocked by the barrier.^[^ ^111^ ^]^

### 8.5 Motion Artifacts and Pressure Disturbance

Pulse oximetry is sensitive to motion artifacts and pressure variations due to any physical activity that can hinder the measurement accuracy of the PPG signals.^[^ ^160^, ^161^ ^]^ Motion artifacts are commonly seen in non‐conformal type oximeter sensors during voluntary or involuntary body movements, which eventually lead to false interpretations of AC and DC components of PPG signals and SpO_2_ measurements. Motion artifacts in general span a broad range of frequencies, ranging from low‐frequency movements to high‐frequency tremors. Low‐frequency artifacts result from slow movements such as voluntary body motions or involuntary muscle contractions. The typical frequency range for low‐frequency aberrations ranges from 0.5 Hz to a few hertz (Hz), while the high‐frequency artifacts can stem from rapid movements, vibrations, or tremors that can reach up to tens of hertz or even higher frequencies. On the other hand, the frequency range of the pulsatile components in the PPG signals ranges from 0.5 Hz to 10 Hz^[^ ^162^, ^163^, ^164^ ^]^ which overlaps with both low‐frequency and high‐frequency noise. Hence, in order to mitigate the impact of motion artifacts and to distinguish between the undesirable motion‐induced noise in PPG signals, oximeters drive the pulsed light sources (red and NIR) that are synchronized with light acquisition from the photodiode in finite temporal window. This drastically reduces additional noises and simply improves the signal‐to‐noise ratio. Nonetheless, oximeters may also integrate intelligent algorithms, signal processing, noise filters, and continuous contact impedance measurement techniques to produce quality PPG signals and accurate data^[^ ^165^, ^166^, ^167^, ^168^, ^169^, ^170^, ^171^ ^]^

Further, the pressure imparted by a sensing probe on the sensing location can also cause variations in the magnitude of the recorded PPG signals. This pressure can cause compressional stress, leading to deformations in the arterial geometry and alterations of the obtained PPG signal. An under pressure can cause inadequate contact between the surface of the probe and skin, thereby affecting the AC signal magnitude. AC signal amplitudes also decrease in overpressure conditions, when the arteries get occluded by blood. Hence, an optimal contact pressure determines the maximum pulsatile amplitude.^[^ ^172^, ^173^, ^174^, ^175^ ^]^ Khan et al. studied the reflection‐based oximetry on a forearm with applied external pressure of 0.7 kPa. In this experiment, AC signal magnitudes for red and NIR light showed some changes in the baseline values. However, as the SpO_2_ oxygen saturation is evaluated as the ratio of AC to DC signal magnitudes at two wavelengths, the ratio of AC and DC signals remained almost the same and showed $R_{OS}^{\prime }=0.65$ with external pressure, and $R_{OS}^{\prime }=0.67$ without external pressure.^[^ ^94^ ^]^

### 8.6 Skin Pigmentation

The effect of skin color on the accuracy of oxygen saturation values measured by pulse oximeters is a topic of discussion for many years as the PPG signals in the pulse oximeter are strongly influenced by the content of melanin in the skin. Indeed, melanin has the ability to absorb light in visible and near‐infrared regions. Melanin is distributed in the skin as structures called melanosomes, produced by melanocyte cells. In dark skin, melanosomes are larger and more numerous and thus absorb more light than the surrounding tissues resulting in an overestimation in the PPG signals readings. Further, the reduced transmission of light through the skin due to absorption by melanin will result in a decrease in PPG signal intensity, precision, and increase in noise. In such a condition, for a dark‐skinned patient suffering from hypoxemia, the oxygen saturation measured will be higher than the actual value, thus disguising the actual health circumstances leading to delayed medical attention.^[^ ^176^, ^177^, ^178^, ^179^, ^180^, ^181^ ^]^

### 8.7 Presence of Dyshemoglobins

Alongside Hb and HbO_2_, adult blood also contains dyshemoglobins such as methemoglobin (MetHb) and carboxyhemoglobin (COHb), which are derivatives of hemoglobin.^[^ ^182^ ^]^ In a healthy individual, these derivatives of Hb are normally present in low concentrations in the blood. However, an increase in dyshemoglobins concentration may reduce the accuracy of SpO_2_ measurement values, since MetHb and COHb also absorb light in the same wavelength range as used in oximetry.^[^ ^183^ ^]^ For example, in patients diagnosed with an adverse health condition such as Haemoglobin Rothschild (a medical condition that results in low arterial oxygen saturation due to a mutation in the haemoglobinβ chain), higher concentration of dyshemoglobins in blood volume usually generates less accurate SpO_2_ data.^[^ ^184^, ^185^ ^]^

### 8.8 Low Blood Oxygen Levels

Low blood oxygen levels naturally result in low SpO_2_ values, for instance, in adverse health conditions such as hypoxia or hypoxemia, where the blood oxygen level is low and the oxygen saturation levels will drop substantially.^[^ ^186^, ^187^, ^188^ ^]^ In another instance, changes in the PPG signal magnitudes measured in sensing locations such as earlobes and fingers that contribute to local body heat transfer mechanisms could be due to variations in the blood perfusion, resulting in low oxygen levels. Under normal conditions, blood flow to the fingers and earlobes is usually higher, leading to a higher SNR in PPG signals from these locations. However, in conditions such as hypothermia (loss of heat from the body) and hyperthermia (heat gain in the body), blood flow to these sensing locations is regulated by the nervous system, depending on the mode of heat transfer. In hypothermia conditions, when the surrounding temperature is colder, the arteries in these sensing locations constrict, causing low blood perfusion to reduce heat dissipation. Consequently, transmission mode oximetry performed on earlobes or fingers in such a condition will likely yield less accurate SpO_2_ data.^[^ ^19^ ^]^

## 9 Components Limiting the Form Factor of Oximeters

At present, one of the major limitations in the realization of fully‐flexible pulse oximeters is the incorporation of analog front‐end (AFE) components^[^ ^95^, ^189^ ^]^ which limits the capability of organic sensors to benefit from their form factor. The form factor of a sensor refers to its physical shape, size, and flexibility. In order to ensure comfortable wearability to users, organic PPG sensors are made thin, lightweight, and body conformable. AFE components amplify, process, and drive the electrical signals acquisition from OPDs. However, AFE components in the current reported organic PPG sensors are made up of traditional inorganic material such as silicon owing to its superior performance qualities such as high signal processing capabilities and fast response times.^[^ ^149^ ^]^

Integration of inorganic AFE components with organic PPG sensors poses difficulties in terms of form factor minimization. However, with the integration of bulky inorganic AFE components, the flexibility properties of organic PPG sensors are hampered as the AFE components often require additional packaging and interconnections.^[^ ^125^ ^]^ Thus, to overcome this limitation, researchers are actively seeking for new alternate approaches to develop organic ppg sensors with integrated fully‐flexible AFE components to enable a more compact and flexible form factor for the sensors. Far from achieving a fully‐organic AFE realization, one first strategy starts with the development of organic analog amplifiers in a CMOS architecture.^[^ ^190^, ^191^, ^192^, ^193^, ^194^, ^195^, ^196^ ^]^ Moreover, these organic components could be developed from solution processing techniques such as printing thus enabling their integration directly onto their substrate without the need for additional packaging.^[^ ^197^, ^198^, ^199^ ^]^ With this approach we believe the form factor of the organic PPG sensors could be drastically reduced.

## 10 Conclusion

Flexible pulse oximetry sensors for biosignal monitoring are a rapidly evolving field that can upgrade the accessibility and quality of healthcare. With active research and wide commercial opportunities, SpO_2_ sensors are expected to replace their rigid counterparts in the future for smart health monitoring. Unlike conventional rigid inorganic pulse oximeter sensors for monitoring SpO2, flexible and organic sensors have unrivaled advantages in terms of conformability, wearability, and continuous real‐time data availability. In this review article, we highlight the recent progress and significance of flexible and wearable organic pulse oximeters in health monitoring and focus on geometry, processing techniques, materials, performance parameters, encapsulation techniques, factors that affect PPG signals, and limitations.

The optimal geometry for OLEDs and OPDs, with optimal spacing distance, should be taken into consideration for better data output and readability with minimum noise. For PPG sensors working in the reflection mode, circular geometry is considered the most effective when compared with rectangular or bracket geometry. As OLEDs and OPDs constitute the heart of the organic PPG sensor architecture, careful consideration in the choice of materials and efficient fabrication techniques for OLEDs and OPDs needs to be employed. Despite extensive research, significant challenges remain in the development of organic SpO_2_ sensors, including poor conformability, accurate measurements, and skin‐contact‐impeding factors. To enable continuous data monitoring, suitable sensor locations should be chosen to obtain the best PPG signal response. Conformal contact between the pulse oximeter and skin will enhance the SNR, so the substrate should be ultrathin and flexible. Nonconformal contact between the skin and the electronic interface not only induces motion artifacts during measurements but can also cause direct coupling of light from the OLED into the OPD, resulting in low SNR. In addition, motion artifacts could cause multiple light interference and scattering effects. To minimize motion artifacts, thin substrates that can conformally adhere to human skin should be used, and signal dependency on motion artifacts should be minimized by careful implementation of intelligent algorithms. Prior knowledge of a patient's health status such as whether they have any cardiovascular diseases or excess dyshemoglobins and understanding the skin pigmentation conditions before measurements, both can avoid false interpretation and evaluation of SpO_2_ data. In addition to the above‐mentioned factors, organic PPG sensors need regular calibration in order to maintain accuracy, because of the inherent variability of organic semiconductor polymer materials and their susceptibility to environmental variables, such as moisture and humidity fluctuations.

Recent progress in organic semiconductor materials and optoelectronics has made it possible to design and fabricate organic and skin‐conformal SpO_2_ sensors. However, the unsatisfying long‐term ambient stability of SpO_2_ sensors must be improved with suitable encapsulation techniques without affecting their functionality when laminated onto flexible skin. Further, research and development initiatives should focus on developing organic AFE components for the realization of all organic PPG sensors. The incorporation of intelligent algorithms for data extraction and processing can also help to accurately evaluate human health conditions. Pulse oximeters can be further improved by incorporating efficient machine learning and large data analysis algorithms to meet long‐term user‐friendly requirements.

In general, SpO_2_ sensors offer an open space for plenty of exciting research contributions for skin‐interfaced medical research. With plenty of exciting opportunities, the future of pulse oximetry sensors is promising. Moreover, empowering users with real‐time health status conditions can enable them to proactively take precautions and follow necessary steps based on the severity of symptoms thus enabling preventive healthcare practices in the future. Thus, we believe that the incorporation of novel polymer semiconductor materials, efficient fabrication techniques, biocompatible designs, novel encapsulation materials and techniques, communication technologies, and incorporation of organic material‐based electronic components will play a significant role in realizing efficient and conformal all organic pulse oximeter sensor for personalized healthcare.

## Conflict of Interest

The authors declare no conflict of interest.
